# Recent Advances in Supramolecular-Macrocycle-Based Nanomaterials in Cancer Treatment

**DOI:** 10.3390/molecules28031241

**Published:** 2023-01-27

**Authors:** Zheng Pan, Xinzhi Zhao, Qiushi Li, Zhanzhan Zhang, Yang Liu

**Affiliations:** 1 Key Laboratory of Functional Polymer Materials (Ministry of Education), College of Chemistry, Nankai University, Tianjin 300071, China; 2 State Key Laboratory of Medicinal Chemical Biology, College of Chemistry, Nankai University, Tianjin 300071, China; 3 School of Medical Imaging, Tianjin Medical University, Tianjin 300203, China

**Keywords:** cancer, macrocycles, host–guest interactions, nanomaterials, supramolecular chemistry

## Abstract

Cancer is a severe threat to human life. Recently, various therapeutic strategies, such as chemotherapy, photodynamic therapy, and combination therapy have been extensively applied in cancer treatment. However, the clinical benefits of these therapeutics still need improvement. In recent years, supramolecular chemistry based on host–guest interactions has attracted increasing attention in biomedical applications to address these issues. In this review, we present the properties of the major macrocyclic molecules and the stimulus–response strategies used for the controlled release of therapeutic agents. Finally, the applications of supramolecular-macrocycle-based nanomaterials in cancer therapy are reviewed, and the existing challenges and prospects are discussed.

## 1. Introduction

Supramolecular chemistry has developed rapidly since Donald J. Cram, Jean-Marie Lehn, and Charles J. Pedersen won the Nobel Prize in 1987 for their contributions to host–guest systems. Supramolecular systems are assembled by non-covalent interactions such as hydrogen bonds, coordination bonds, hydrophobic interactions, electrostatic interactions, and π-π stacking [1,2]. Compared with covalent interaction, non-covalent interactions demonstrate several advantages. First, non-covalent interactions offer a facile strategy for constructing supramolecular materials, effectively avoiding complicated synthesis and purification processes [3]. Such supramolecular-based strategies are generally environmentally friendly and cost/time-saving, since supramolecular materials are readily prepared by simply mixing functional units in solution under ambient conditions [4]. In addition, this non-covalent interaction endows supramolecular materials with dynamic and reversible properties, especially the ability to respond to external stimuli, which provides great potential for the design and construction of stimulus–response materials based on supramolecular chemistry [5]. Most importantly, supramolecular chemistry allows for the manipulation of functional units at the molecular level, enabling a “bottom-up” strategy to control the size and morphology of supramolecular materials. In particular, the construction of supramolecular materials with uniform sizes in the nanometer range has attracted increasing attention in biomedical applications.

Among various non-covalent interactions, host–guest interactions have received increasing attention in biomedical applications by integrating macrocyclic molecules into supramolecular materials. During the past few decades, a series of macrocyclic hosts, including crown ethers, cyclodextrins, calix(n)arenes, cucurbit(n)urils, and pillar(n)arenes, have been developed [6,7,8,9]. Typically, these macrocyclic hosts possess a hydrophobic cavity and a hydrophilic outer shell, allowing the accommodation of guest molecules into their cavities. This unique host–guest interaction provides molecular-level encapsulation for drug molecules, thereby effectively protecting drug molecules from degradation or inactivation. In addition, most host–guest interactions have a host–guest stoichiometry of 1:1 at thermodynamic equilibrium and are determined by the characteristic constant of association (Ka) [10]. As a result, the drug loading content can be directly predicted at a given concentration and association constant. 

Taking advantage of host–guest interactions, several limitations that hinder the clinical application of anticancer drugs can be effectively addressed. For example, the solubility and stability of anticancer drugs can be effectively improved by forming host–guest complexes with macrocyclic hosts [11,12]. In addition, a high accumulation of therapeutic agents can be guaranteed by forming supramolecular self-assembly, resulting in significantly enhanced therapeutic efficacy and reduced toxic effects [13]. Furthermore, the upper and lower rim of the macrocyclic hosts are easily modified to incorporate functional groups—for example, imaging agents, targeting ligands, and even therapeutic agents—thus endowing these macrocyclic hosts with therapeutic properties [14,15]. More importantly, the dynamic binding nature of host–guest interaction allows the macrocyclic host to precisely control the release of loaded cargo in response to abnormal biomarkers in tumor tissues—for example, acidic pH, GSH, ATP, and hypoxic. The dynamic nature of host–guest interactions makes macrocyclic host-based anticancer strategies more versatile than traditional nanomedicines that lack stimulus responsiveness.

**Figure 1:**
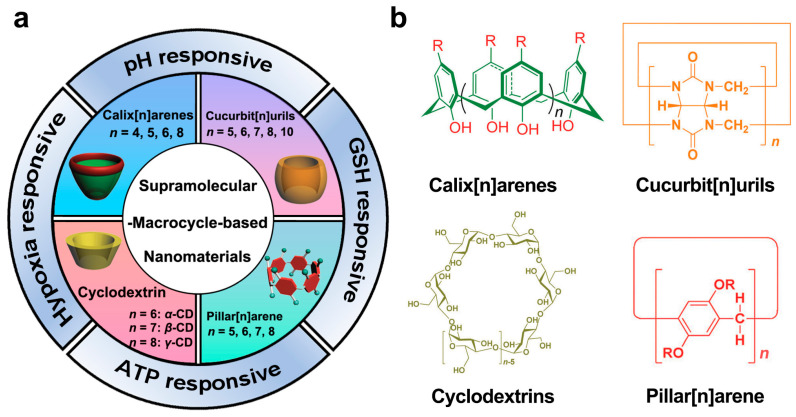
(**a**) Recent advances in supramolecular-macrocycle-based nanomaterials in cancer treatment. (**b**) The chemical structure of four major macrocyclic molecules. Reproduced from ref. [6]. Copyright 2017 Royal Society of Chemistry.

This review introduces the properties of four major macrocyclic hosts, including cyclodextrins, calixarenes, pillararene, and cucurbiturils (Figure 1). Next, strategies for the controlled release of therapeutic agents based on stimulus–response strategies (hypoxic, acidic, GSH, and ATP) are demonstrated. Finally, we summarize the recent advances in supramolecular-macrocycle-based nanomaterials for cancer treatment, including supramolecular-macrocycle-based chemotherapy, supramolecular-macrocycle-based photodynamic therapy, and supramolecular-macrocycle-based combination therapy [16,17]. 

## 2. Macrocyclic Host Molecules

Poor solubility and stability are two major factors restricting the general applications of 40–70% of newly developed anticancer drugs/probes in clinical cancer treatment. Encapsulating therapeutic agents into macrocyclic hosts offers a feasible strategy to address these issues. This section discusses four major types of macrocyclic hosts for fabricating supramolecular therapeutic agents, including cyclodextrins, calixarenes, and cucurbiturils.

### 2.1. Cyclodextrin

Cyclodextrins (CDs) have received increasing attention among the many macrocyclic host molecules, especially for their biological applications. CDs refer to a class of water-soluble macrocyclic oligosaccharides linked by multiple α-1,4 glycosidic bonds [18]. Since the French scientist Villiers accidentally discovered CDs in natural products in 1891, it has undergone hundreds of years of development [19]. Moreover, the easy accessibility of CDs from starch precursors such as corn, rice, and potato also facilitated its development [20]. To date, CDs have been extensively applied in various fields, including pharmaceutics, catalytic reactions, enzyme technology, and analytical chemistry [21,22,23]. The commercially available CDs have three major subtypes, γ, β, and α-CD, consisting of eight, seven, and six D-glucose units. CDs have a truncated cone-like molecular container structure with a hydrophilic external surface and a hydrophobic interior cavity. This unique structure enables CD to form host–guest complexes with various guest molecules, such as drugs, amino acid residues, and fluorescent probes, in an aqueous solution through van der Waals interaction, hydrogen binding, and hydrophobic interactions [24,25]. The resulting host–guest complex effectively improves the safety, stability, and solubility of the loaded drugs, thereby significantly reducing side effects and enhancing bioavailability [26,27]. In addition, the saccharide nature of CDs determines their excellent biocompatibility. Benefiting from these unique properties, more than 54 CD-based nanomedicines and prodrugs have been developed and applied in clinical and preclinical studies.

### 2.2. Calix(n)arenes

Calix(n)arenes (CAs), representing the third generation of macrocyclic hosts after CDs and crown ethers, are typically prepared by bridging multiple phenolic units with methylene groups at the 2- and 6-positions [28]. The CAs have a corn-like structure, with a hollow cavity and two rims on the upper and lower sides [29]. Due to the fully synthetic process, CAs exhibit several advantages over naturally available CDs, including ease of modification, controllable conformations, and tunable scaffolds (usually composed of 4, 5, 6, or 8 units) [30]. With these characteristics, CAs are considered macrocycle hosts with unlimited structure and possibilities [31]. As potential therapeutic modifiers and drug carriers, the water-soluble CAs were synthesized by introducing functional groups such as sulfonic acid, carboxylic acid, amino, or quaternary ammonium to the upper or lower rim of CAs [32,33]. Like CDs, CAs can load guest molecules such as drugs and fluorescent probes through host–guest interactions [34,35,36]. More importantly, their binding affinities can be adjusted in response to abnormal biomarkers in tumor tissues, allowing for precise control of drug release in tumor tissues.

### 2.3. Cucurbit(n)urils

Compared with CDs and CAs, the cucurbit(n)urils (CBs) have a relatively short history. As early as 1905, Behrend et al. reported the synthesis of CBs by condensing glycoluril and formaldehyde. However, it was only in 1981 that the Mock group gave the definite chemical structure of CBs, and finally, Kim et al. isolated CB members around 2000 [37]. CBs have a highly symmetric pumpkin-like structure with a central hydrophobic cavity and two hydrophilic carbonyl rims. The CB family possesses a uniform cavity depth of 0.91 nm, and the width depends on the number of glycoluril units [38,39]. Uniquely, the cavity of CBs is hydrophobic and nonpolar due to the absence of lone pairs of electrons or chemical bonds within the cavity [40]. Therefore, CBs are ideal macrocyclic hosts for encapsulating neutral and positively charged guest molecules. For example, CB(6) can host alkyl ammonium ions, while CB(7) can host adamantanamine. CB(8) is even capable of hosting two guest molecules (2,6-bis(4,5-diydro-1H-imidazol-2-yl)naphthalene) by forming 1:2 host–guest complexes [41]. Despite their extremely high binding affinities toward various guest molecules, the low water solubility of CBs is a significant bottleneck that limits their general application. To address these issues, functionalized CBs were developed by using aldehyde or glycoluril derivatives in the condensation process [42,43]. Although promising, the complicated synthesis and purification procedures still limit their applications. Therefore, developing straightforward and efficient functionalization strategies is crucial for CB’s applications.

### 2.4. Pillar(n)arene

Similar to CBs, pillar(n)arene (PAs) also have a short history. In 2008, Ogoshi synthesized a type of phenol para-bridged “pillar” supramolecular macrocyclic molecule called pillararenes [44]. Pillararenes have a rigid pillar structure and hydrophobic cavities. The size of the cavities can be regulated by changing the number of repeating units, which can be named pillar(n)arene (*n* = 5, 6, 7, 8) [6]. Moreover, water-soluble pillar(n)arene, such as water-soluble pillar(5)arene (WP5) and water-soluble pillar(6)arene (WP6), were developed through functional modification [45,46]. Similarly to CDs and CAs, pillar(n)arenes have excellent host–guest recognition ability to achieve effective loading of guest molecules [47]. Moreover, due to its electron-rich cavities and polyhydroxy or alkoxy structures, pillar(n)arene can form supramolecular complexes with pyridine salts, ferrocene, and quaternary ammonium salts through non-covalent interactions, which broadens the variety of host–guest complexes [48,49,50]. Benefiting from these unique properties, pillar(n)arene has attracted increasing attention in biomedical applications.

## 3. Strategies for Controlled Drug Release

The abnormal proliferation and metabolism of tumor cells lead to overexpressed physiological indicators in TME. In addition, the dynamic binding nature of host–guest interaction offers great potential for the macrocyclic host to respond to these indicators to release loaded cargo in tumor tissues. This section will briefly introduce the recently developed stimulus–response supramolecular-macrocycle-based nanomaterials for cancer treatment based on acidity, redox, ATP, and hypoxia conditions.

### 3.1. pH-Responsive Supramolecular-Macrocycle-Based Nanomaterials

Acidity is a typical hallmark for distinguishing tumors from normal tissues, since most solid tumors are accompanied by excessive accumulation of acidic metabolic organelles in the TME [51]. Many pH-responsive macrocyclic hosts have been developed for tumor-targeted delivery of anticancer drugs, which is mainly achieved by introducing ionizable functional groups into macrocyclic hosts [52,53]. In acidic TME, the physical properties, cavity size, and chemical structure of these functionalized macrocyclic hosts are changed due to the protonation effect, resulting in a significant decrease in binding affinity and the on-demand release of loaded drugs in the TME. For example, Li et al. presented an N, N-diisopropylenediamine (DPA)-grafted β-CD (β-CD-DPA) to deliver succinobucol (SCB) for the effective treatment of breast cancer [54]. SCB was loaded into the hydrophobic domain of β-CD-DPA. Upon reaching tumor tissue, DPA efficiently protonated and transformed β-CD-DPA to a hydrophilic state, leading to the release of SCB and tumor suppression in the 4T1 breast cancer mouse model. Similarly, Li et al. reported a carboxylatopillar(6)arene (CP6A) for the tumor-targeted delivery of oxaliplatin (OX) [55]. Under acidic conditions, the binding affinity of CP6A to OX was significantly reduced due to the protonation effect, thereby achieving the controlled release of OX in tumor tissues. In addition, introducing pH-responsive motifs in guest molecules is also a promising strategy for constructing pH-responsive supramolecular-macrocycle-based nanomaterials. For example, Liu et al. reported a pH-responsive double positive charged guest molecule, ADA2+ [56]. They constructed a supramolecular nanoparticle (ADA2+@HACD) by complexing with hyaluronic acid-modified β-CD (HACD) for efficient plasmid DNA (pDNA) delivery. The strong positive charge of ADA2+ enabled ADA2+@HACD to condense and encapsulate pDNA efficiently. Under the acidic conditions, the ester bonds of ADA2+ were degraded to carboxyl groups, resulting in the controlled release of loaded pDNA in tumor tissues.

### 3.2. GSH-Responsive Supramolecular-Macrocycle-Based Nanomaterials

GSH is one of the essential biomarkers in tumor tissues and is closely related to the occurrence and progression of tumors. In addition, the significantly different levels of GSH in extracellular space (1–10 μM) and inside cells (2–10 mM) make GSH an ideal and ubiquitous trigger for intracellular drug delivery [57,58]. For example, Wang et al. constructed a β-D-galactose-modified pillar(5)arene (GlaP5) for tumor-targeted delivery of camptothecin (CPT) [59]. Firstly, CPT was prepared as a prodrug by introducing trimethylammonium groups and disulfide bonds. The trimethylammonium groups function as the binding sites to efficiently deliver CPT prodrugs, and the disulfide bonds function as responsive units to allow the controlled release of CPT in tumor tissues. With this strategy, they greatly enhanced the cytotoxicity of CPT against HepG2 liver cancer cells. Xu et al. reported a GSH-responsive poly-cyclodextrin nanocage (PDOP NCs) to deliver doxorubicin (DOX) for enhanced cancer immunotherapy [60]. After systemic administration, PDOP NCs accumulated in tumor tissues via the enhanced permeability and retention (EPR) effect. Subsequently, DOX was released from PDOP NCs to activate immunogenic cell death (ICD) of 4T1 cells, thereby enhancing cancer immunotherapy. Aside from disulfide bonds, the ferrocenium cation is another commonly used functional group to construct GSH-responsive drug delivery systems. For example, Pei et al. reported a novel ferrocenium-capped pillar(5)arene (FCAP) to deliver siRNA and DOX [61]. Driven by iron ions, FCAP self-assembles into nanoparticles to facilitate the cellular uptake of DOX and siRNA. After internalization, the ferrocene cation was efficiently reduced to neutral ferrocene by GSH, leading to the disassembly of the nanoparticles and the release of the loaded cargoes. Similarly, Zhu et al. reported a ferrocenium-integrated supramolecular block polymer (SBC) and achieved intracellular delivery of pDNA with high transfection efficiency [62].

### 3.3. ATP-Responsive Supramolecular-Macrocycle-Based Nanomaterials

ATP is an essential compound for life, providing energy for most processes in living systems, such as chemical synthesis, dissolution of condensates, transmission of nerve impulses, and muscle contraction [63,64,65]. In particular, ATP is significantly elevated in tumor tissues due to its rapid proliferation and metabolism [66]. Recently, several ATP-responsive supramolecular-macrocycle-based nanomaterials have been developed for tumor diagnosis and targeted delivery. For example, Guo et al. presented a novel amphiphilic guanidinium-modified calix(5)arene (GC5A-12C) for tumor-targeted delivery of photosensitizers (PS) [67]. GC5A-12C is designed to contain multiple guanidine groups on its upper rim, which allows GC5A-12C to form a salt bridge with ATP/PS through electrostatic interactions and hydrogen bonds. During blood circulation, the strong binding affinity between GC5A-12C and PS effectively quenches PS fluorescence and avoids payload leakage. Upon reaching the tumor tissues, the PS is outcompeted by overexpressed ATP, accompanied by restoration of fluorescence and photoactivity. Similarly, Meng et al. presented a cationic water-soluble pillar(6)arene (WP6A)-based ATP-responsive supramolecular drug delivery system for tumor-targeted delivery of DOX [68]. In tumor tissue, a high concentration of ATP acts as the competitive guest molecule. It bound to WP6A competitively, leading to the disassembly of the supramolecular assembly and the release of DOX. In addition to being used as a biomarker for designing responsive nanomaterials, capturing ATP offers an alternative strategy for cancer treatment. For example, Huang et al. reported a trimethylammonium-modified pillar(6)arene (WP6A), which recognized and exhausted intracellular ATP to block the energy supply [69]. As a result, WP6A effectively overcame drug resistance and significantly enhanced the cytotoxicity of DOX on MCF-7/ADR cells.

### 3.4. Hypoxia-Responsive Supramolecular-Macrocycle-Based Nanomaterials

Hypoxia is another indicator of tumors. Tumor hypoxia is usually caused by abnormal angiogenesis and rapid proliferation of tumor cells [70]. The lack of oxygen in the tumor site causes an imbalance in the redox state of the cancer cells [71]. Taking advantage of this property, Guo et al. introduced azo groups into the upper rim of calixarenes and designed a series of hypoxia-responsive azocalixarenes [72,73,74]. For example, they reported a carboxylated azocalix(4)arene (CAC4A) for hypoxia-targeted drug delivery [75]. CAC4A exhibited a strong binding affinity to various anticancer drugs during blood circulation, effectively avoiding payload leakage and side effects. Upon reaching the hypoxic TME, the azo groups of CAC4A were effectively reduced by bio-reductase, resulting in significantly decreased binding affinity and the release of drugs in tumor tissues. Additionally, the same group also developed sulfonated azocalix(5)arene (SAC5A) and achieved tumor-targeted delivery of paclitaxel (PTX) [76]. By integrating macrocyclic hosts in nanosystems, our group reported a macrocyclic-amphiphile-based self-assembled nanoparticle (MASN) for ratiometric delivery of drug combinations to tumor tissues [77]. In hypoxic TME, MASN was reduced by bio-reductase, leading to the spontaneous release of drug combinations in tumor tissues. Through precise loading and ratiometric co-delivery of drug combinations, MASN achieved effective combination chemotherapy and significantly suppressed tumor growth in a 4T1 breast cancer mouse model. Similarly, we developed a calixarene-integrated nano-drug delivery system (CanD) and achieved tumor-targeted delivery and tracking of anticancer drugs in vivo [67].

## 4. Supramolecular-Macrocycle-Based Nanomaterials for Enhanced Chemotherapy

Chemotherapy refers to the use of cytotoxic drugs to control and kill tumors. Despite the remarkable success, common issues associated with low bioavailability and high cytotoxicity of anticancer drugs still limit their general applications in clinical cancer treatment [78,79]. In addition, increased interstitial fluid pressure (IFP) also limits the penetration of anticancer drugs into deep tumor tissues, further restricting the therapeutic efficacy of anticancer drugs [80]. Compared with traditional nanocarriers, supramolecular-macrocycle-based nanomaterials possess the following advantages: (i) precise loading and on-demand release of drugs; (ii) easy size manipulation at tumor sites due to the dynamic nature of host–guest interactions [29,81]. With these properties, supramolecular-macrocycle-based nanomaterials demonstrate great potential to improve the therapeutic efficacy of anticancer drugs by enhancing tumor accumulation and promoting tumor penetration. This chapter mainly discusses the progress of supramolecular-macrocycle-based nanomaterials in these aspects.

### 4.1. Enhancing Tumor Accumulation

**Figure 2:**
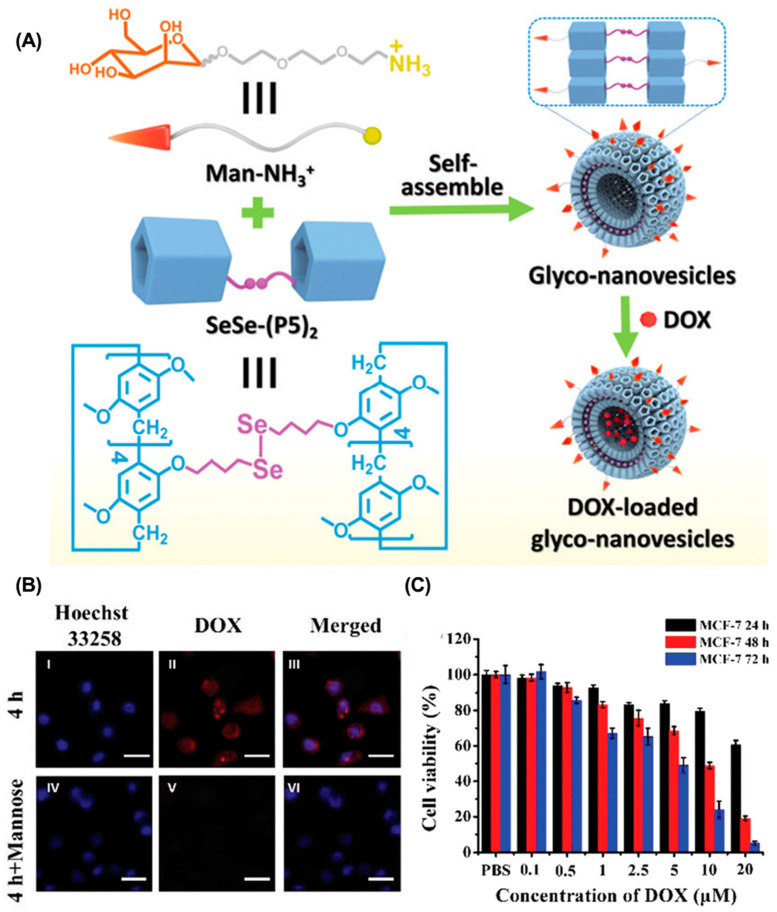
Glycol-NV for targeted drug delivery to enhance tumor accumulation. (**A**) Schematic illustration of the construction of glycol-NV. (**B**) CLSM of MCF-7 cells incubated with glycol-NV with or without pre-incubation of mannose. (**C**) Cell viability of MCF-7 cells incubated with glycol-NV. Reproduced from ref. [86]. Copyright 2020 Royal Society of Chemistry.

**Figure 3:**
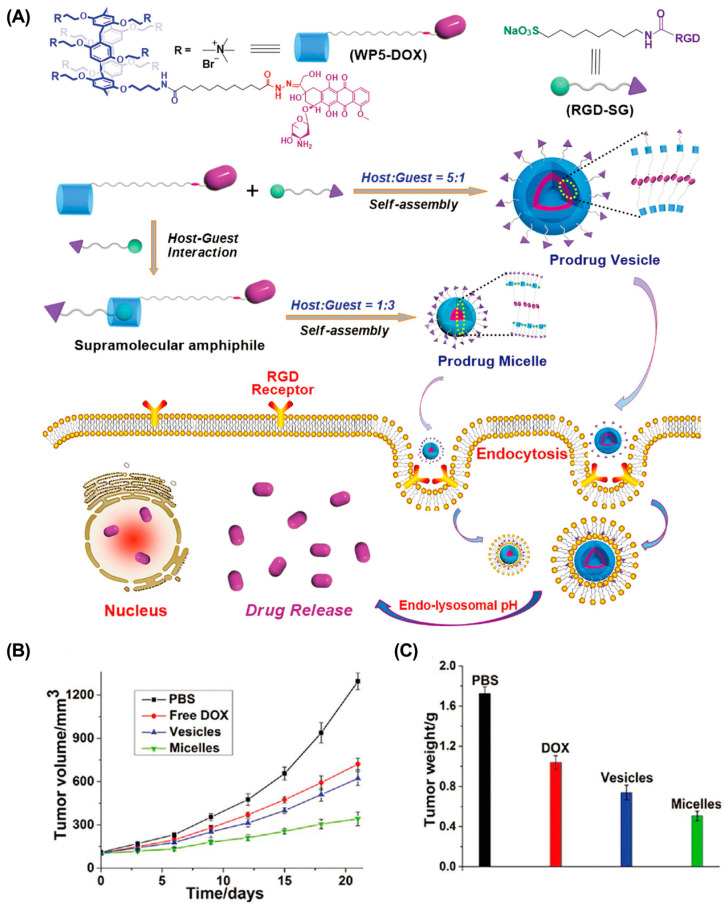
RGD-modified size-controllable supramolecular nanocarriers for targeted drug delivery. (**A**) Schematic illustration of the construction of supramolecular vesicles and micelles. (**B**) Tumor volume and (**C**) tumor weight of different treated groups. Reproduced from ref. [88]. Copyright 2018 Wiley-VCH.

Compared with conventional anticancer drugs, nanomedicines have relatively controllable biodistribution and can passively accumulate at tumor sites through the EPR effects [82]. In addition, active tumor targeting is also feasible by introducing specific targeting ligands. The mutation or overexpression of glycoproteins on the cell surface is a typical class of tumor markers–for example, carbohydrate antigen (CA125), CAl5-3, and CA50 [83,84,85]. Modifying the corresponding glycosylated ligands in drug delivery systems is an effective strategy for active tumor targeting. For example, Pei et al. constructed a mannose-modified GSH-responsive supramolecular vesicle (glycol-NV) for tumor-targeted delivery of DOX (Figure 2, Table 1) [86]. The glycol-NV was formed by self-assembly of mannose derivatives (Man-NH3+) and diselenium-bridged pillar(5)arene dimers (SeSe-(P5)2). The decorated mannose effectively guided glycol-NV to tumor cells through the interactions between mannose and mannose receptors. After cellular internalization, overexpressed GSH led to the cleavage of the Se-Se bonds in glycol-NV, resulting in the efficient release of DOX and enhanced antitumor efficacy. Similarly, Pei et al. presented a galactose-integrated supramolecular vesicle (CAAP5G) for systemic delivery of DOX and achieved significantly enhanced suppressions in HpeG2 cells [87]. Aside from glycosylated ligands, antigens and peptides are also commonly used as targeting ligands. For example, the tripeptide Arg-Gly-Asp (RGD) binds strongly to integrin αvβ3, which is overexpressed on the surface of various cancer cells, including melanoma, breast, and ovarian cancer. Taking advantage of RGD, Schmuck et al. reported size-controllable supramolecular nanocarriers (WP5-DOX⊃RGD-SG) for targeted delivery of DOX (Figure 3) [88]. By adjusting the host–guest molar ratio, WP5-DOX⊃RGD-SG was assembled into both vesicles and micelles, and it was found that the micellar WP5-DOX⊃RGD-SG demonstrated significantly enhanced antitumor efficacy in a HepG 2 liver cancer mouse model. Similarly, Zhang et al. developed an RGD-modified layer-by-layer film based on CD and Ada interactions for tumor-targeted delivery of DOX, and achieved significantly enhanced antitumor efficacy in an A549 tumor mouse model [89].

### 4.2. Enhancing Tumor Penetration

**Figure 4:**
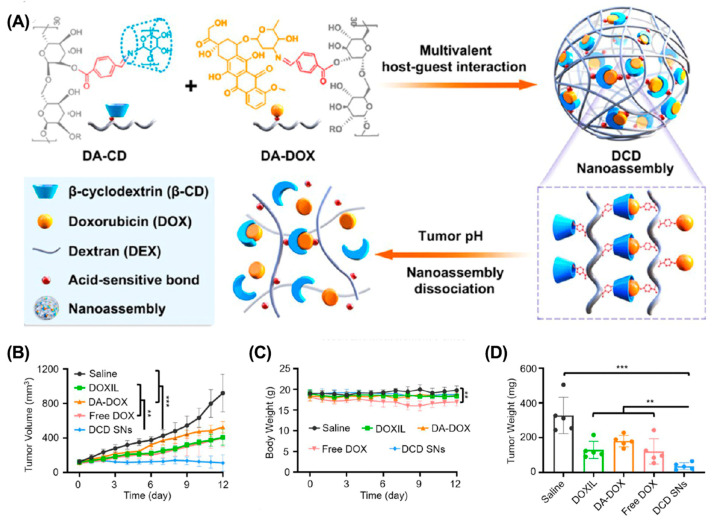
DCD SN for deep tumor penetration to enhance cancer therapy. (**A**) Schematic illustration of the construction of DCD SNs. (**B**) Tumor growth curves, (**C**) body weight, and (**D**) tumor weight after different treatments. (** *p* < 0.01, *** *p* < 0.001). Reproduced from ref. [96]. Copyright 2021 American Chemical Society.

Although various strategies effectively enhance drug accumulation in tumors, their clinical benefits are still unsatisfactory. The inherent properties of solid tumors, including the dense extracellular matrix and high IFP, make it difficult for large-sized nanoparticles to penetrate deeply into the tumor tissues [90,91]. Therefore, constructing small-sized nanoparticles is a promising strategy to enhance tumor penetration. However, small-sized nanoparticles may undergo rapid clearance by the reticuloendothelial system (RES) during blood circulation [92]. Recently, several size-adjustable supramolecular-macrocycle-based drug delivery systems have been developed to solve this dilemma [93,94,95]. These nanoparticles usually have a relatively large size during blood circulation to ensure efficient tumor accumulation. Upon reaching tumor tissues, they transform into smaller nanoparticles, allowing for efficient tumor penetration. For example, Xu et al. constructed a size-convertible supramolecular nanocomponent (DCD SNs) for efficient tumor penetration (Figure 4) [96]. DCD SNs (126 nm) were prepared by self-assembly of β-CD-modified polyhydroxy dextran (DA-CD) and DOX-modified polyhydroxy dextran (DA-DOX). Upon reaching acidic TME, the DCD SNs dissociate into smaller particles (~30 nm), effectively penetrating deeply into the tumor to enhance chemotherapeutic efficacy. Similarly, Guo et al. constructed a size-switchable supramolecular self-assembly for DOX delivery via host–guest interactions between WP5 and polyethylene glycol-modified aniline tetramer (TAPEG) [97]. They achieved significantly enhanced tumor suppression in a CT26 colon cancer mouse model.

## 5. Supramolecular-Macrocycle-Based Nanomaterials for Enhanced Photodynamic Therapy

Photodynamic therapy (PDT), as a non-invasive, highly selective, and controllable strategy, has attracted more and more attention in cancer treatment [98]. PDT requires three essential elements: light, oxygen, and photosensitizers (PS). PDT employs PS to absorb light, transfer energy to the surrounding oxygen, and generate cytotoxic ROS to destroy proteins, DNA, and lipids in tumor cells, and also induce apoptosis [99]. Although PDT has made significant progress in clinical practice, it suffers from several limitations: (1) the inherent limitations of PS: for example, the aggregation-caused quenching (ACQ) effect and dark toxicity; (2) insufficient oxygen (O2) supply: oxygen, an important PDT element, provides the raw material for ROS generation [100]. However, abnormal neovascularization may lead to insufficient O2 supply, resulting in limited ROS generation and tumor suppression [101]. The rapid development of supramolecular-macrocycle-based nanomaterials offers great opportunities to address these issues in PDT. For example, these nanomaterials can efficiently improve PS’s water solubility and stability, thereby effectively avoiding the ACQ effect and dark toxicity [102,103]. In addition, supramolecular-macrocycle-based nanomaterials can carry O_2_ or O_2_-generation agents, thereby improving O_2_ supply and ROS generation. This section will discuss the recent advances in supramolecular-macrocycle-based nanomaterials for PDT.

### 5.1. Overcome the Inherent Defects of PS

The ACQ effect and dark toxicity are two defects of PS that restrict the general application of PDT. The following describes the application of supramolecular-macrocycle-based nanomaterials in overcoming these limitations for enhanced PDT.

**Figure 5:**
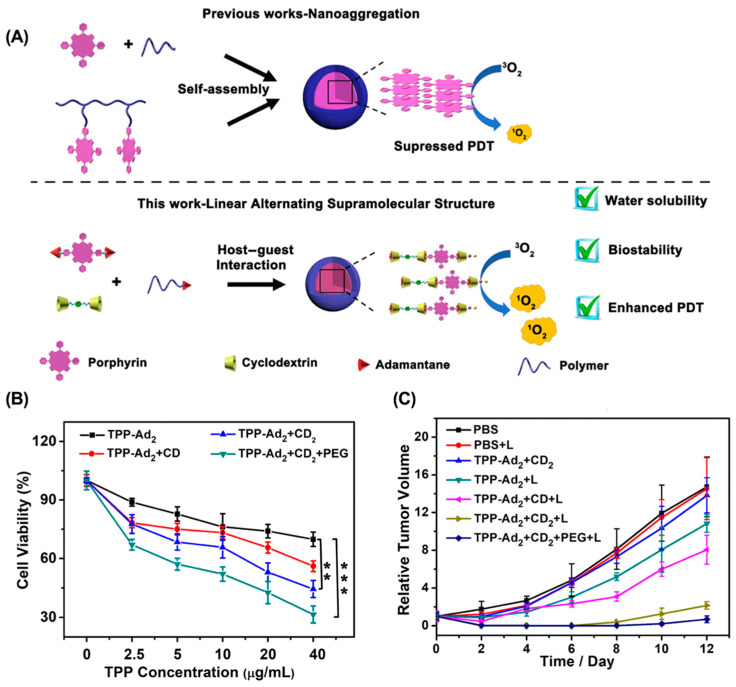
Linear supramolecular polymer for overcoming the ACQ effect to enhance PDT. (**A**) Schematic for the preparation of supramolecular nanoparticles from linear supramolecular polymer. (**B**) In vitro phototoxicity toward 4T1 cells. (**C**) Tumor weight of 4T1 tumor-bearing mice after different treatments. (** *p* < 0.01, *** *p* < 0.005). Reproduced from ref. [107]. Copyright 2020 American Chemical Society.

**ACQ effect.** ACQ refers to the unique phenomenon in which a fluorophore is highly luminescent in the solution state, but becomes weak or non-luminescent in the aggregated state [104]. However, most PSs are hydrophobic and easily aggregated in an aqueous solution, significantly reducing singlet oxygen generation efficiency [105,106]. Therefore, increasing the water solubility of PS to avoid aggregation is a promising strategy to overcome the ACQ effect of PS and enhance the antitumor efficacy of PDT. For example, Zhang et al. reported a linear supramolecular polymer based on the host–guest interaction between β-CD and AD-modified porphyrin (Figure 5) [107]. The unique linear structure increases the water solubility of the porphyrin, and the introduction of β-CD increases the steric hindrance between PSs, which significantly inhibits the aggregation of the porphyrin and improves the efficacy of PDT. Similarly, introducing β-CD to tetraphenyl porphyrin also overcame the ACQ effect and greatly enhanced tumor suppression in a 4T1 breast cancer mouse model [108].

**Figure 6:**
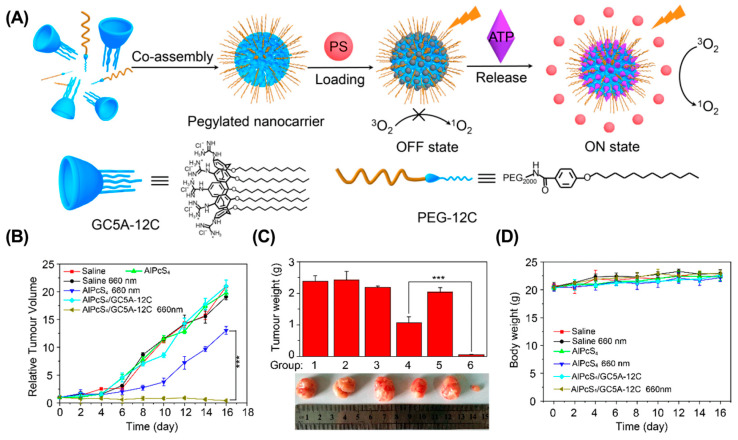
ATP-responsive supramolecular assembly for enhanced PDT. (**A**) Schematic illustration of the preparation of GC5A-12C nanocarrier. The tumor volume (**B**), tumor weight (**C**), and body weight (**D**) in different groups. (*** *p* < 0.001). Reproduced from ref. [111]. Copyright 2018 American Chemical Society.

**Dark toxicity.** Dark toxicity refers to the inherent toxicity of PS without irradiation. The in vivo dark toxicity of PSs is mainly associated with the non-specificity biodistribution of PSs [109,110]. Therefore, optimizing the biodistribution of PSs is a feasible strategy to address this issue. For example, Guo et al. developed an ATP-responsive supramolecular assembly (GC5A-12C) for enhanced PDT (Figure 6) [111]. During blood circulation, the PS was loaded into the cavity of GC5A-12C to avoid undesired dark toxicity, and was competed out by overexpressed ATP as tumor tissues were reached. As a result, GC5A-12C achieved a significantly enhanced antitumor effect in the 4T1 breast cancer mouse model without causing noticeable toxic effects. Similarly, Tang et al. reported a supramolecular PDT system based on the host–guest interaction between p-sulfonatocalix(4)arene and pyridinium-functionalized tetra phenylethylene, which effectively avoided the dark toxicity of PS and significantly suppressed tumor growth in a 4T1 breast cancer mouse model [112].

### 5.2. Alleviating Tumor Hypoxia

**Figure 7:**
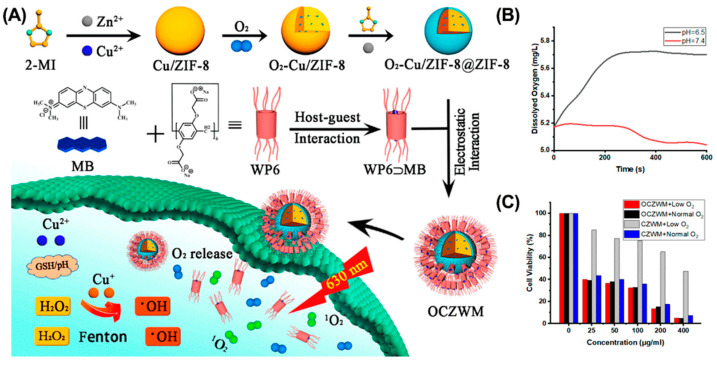
OCZWM for alleviating tumor hypoxia to enhance PDT therapy. (**A**) Schematic illustration of the preparation of OCZWM. (**B**) The O_2_ content under the OCZWM (pH = 6.5 and 7.4). (**C**) Cell viability of HepG2 cells after treatments. Reproduced from ref. [116]. Copyright 2021 Licensee MDPI, Basel, Switzerland.

Oxygen, a critical PDT element, is essential for ROS generation and tumor ablation. However, abnormal proliferation of tumor cells and dysfunctional angiogenesis lead to the formation of a hypoxic microenvironment that significantly limits the efficacy of PDT [70,113]. Recent studies have shown that several types of nanomaterials can interact with, store, and release O_2_ in a controlled manner at tumor sites [113,114,115]. Integrating these nanomaterials into the supramolecular system is a promising strategy to alleviate tumor hypoxia for enhanced PDT. For example, metal–organic framework (MOF) is a porous nanomaterial that can adsorb and transport oxygen. Taking advantage of MOF, Pei et al. reported a zeolitic imidazolate frameworks-8 (ZIF-8)-based supramolecular system (OCZWM) for enhanced PDT (Figure 7) [116]. The OCZWM was formed by coordinating the host–guest complex formed by WP6 and methylene blue (MB) with ZIF-8. After internalization, the OCZWM efficiently decomposes to release loaded MB and O_2_ in response to acid. As a result, OCZWM effectively alleviated tumor hypoxia and significantly enhanced the anti-tumor efficacy of PDT.

**Figure 8:**
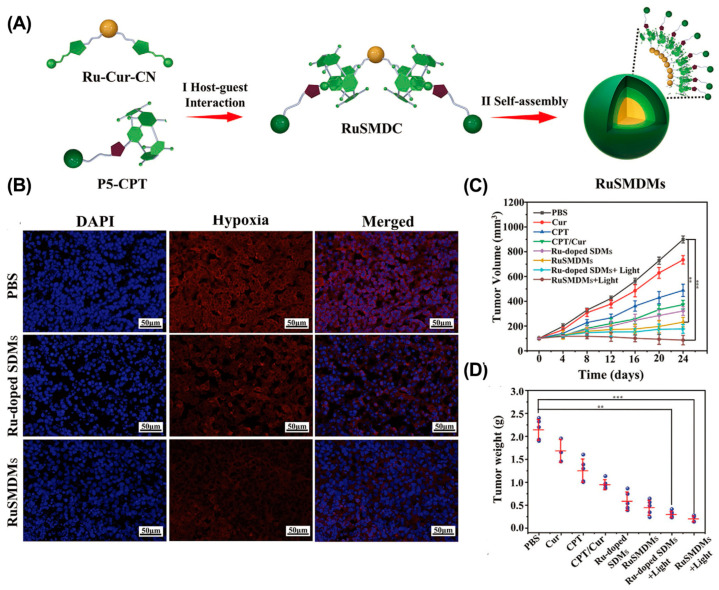
RuSMDC for in situ oxygen supply to enhance PDT therapy. (**A**) Schematic illustration of the preparation of RuSMDC. (**B**) Immunofluorescence imaging of tumor tissue. (**C**) Tumor growth curves. (**D**) Tumor weight in different groups. (** *p* < 0.005; *** *p* < 0.001). Reproduced from ref. [118]. Copyright 2021 Wiley-VCH GmbH.

Although delivering O_2_ directly to the tumor can effectively increase the O_2_ content, O_2_ leakage limits its effectiveness. In situ oxygen generation is an alternative strategy to alleviate tumor hypoxia [117]. For example, Tian et al. reported ruthenium (II)-coordinated supramolecular complexes (RuSMDC) for in situ oxygen production to alleviate tumor hypoxia (Figure 8) [118]. RuSMDC was constructed by host–guest complexing of WP5 with Ru metal complexes. After internalization, Ru catalyzed hydrogen peroxide to generate O_2_, providing sufficient raw materials for ROS generation. In vitro and in vivo experiments showed that RuSMDC effectively alleviated tumor hypoxia and enhanced the efficacy of PDT. Furthermore, the Fenton reaction can also be used to generate O_2_ [119]. For example, Gao et al. reported the β-CD and Ce6-modified Cu_2_–xSe nanoparticles (CS–CD–Ce6 NPs) to alleviate tumor hypoxia via the Fenton reaction [120]. In tumor tissue, Cu+ ions effectively decomposed H_2_O_2_ to O_2_ and •OH, increasing the O_2_ concentration and providing substrates for Ce6 to generate ROS. As a result, the CS–CD–Ce6 NPs effectively alleviated tumor hypoxia and improved the antitumor effect of PDT in a 4T1 cancer mouse model. Similarly, Lu et al. constructed a supramolecular micelle by host–guest complexing β-CD with ferrocene (Fc) [121]. In tumor tissue, Fc is oxidized to Fc+ and catalyzes O_2_ generation, thereby significantly alleviating tumor hypoxia and enhancing PDT therapy.

## 6. Supramolecular-Macrocycle-Based Nanomaterials for Enhanced Combination Therapy

Cancer is one of the most malignant diseases worldwide, involving various genetic alterations and cellular abnormalities and posing significant challenges to traditional monotherapy [122,123]. Common issues associated with monotherapy include low response rate, drug resistance, and side effects [124]. A combination therapy that employs two or more anticancer drugs is a promising strategy to solve these problems. Generally, conventional “cocktail” therapies were achieved by co-administrating multiple drugs [125]. However, different physicochemical properties of these drugs may lead to different pharmacokinetics (PK) and biodistribution in vivo, resulting in limited synergistic effects. The development of the drug delivery system offers great potential to solve these problems by co-loading multiple drugs within a single nanocarrier [126,127]. With this strategy, the PK and biodistribution of combined drugs can be effectively unified, ensuring the synergism of multiple drugs in tumor tissues. In this chapter, we will first introduce the rationale for combination therapies and then discuss the recent advances in supramolecular-macrocycle-based nanomaterials for enhanced combination therapy.

### 6.1. Rationales for Combination Therapy

Combination therapy is meaningful if it produces better clinical outcomes than a single drug [128]. To this end, the choice of drugs for combination therapy is the priority to be considered, which should be based on the following general principles: (1) the selected drugs should have non-overlapping toxicities so that they can be administered at near-maximal doses; (2) the selected drugs should have different mechanisms of action and minimal cross-resistance to avoid the development of multidrug resistance (MDR) [129]; (3) the selected drugs should be demonstrated to have synergistic or additive anticancer efficacy relative to single drugs, which is currently quantified by the Chou–Talalay method. In the Chou–Talalay method, the synergistic effect of drug combinations is usually expressed in terms of combination index (CI), in which CI < 0.9 represents synergism, 0.9 < CI < 1.1 represents additive effect, and CI > 1.1 represents antagonism [130].

### 6.2. Supramolecular-Macrocycle-Based Combination Therapy

Combination therapy can be roughly divided into three categories according to the molecule nature of anticancer therapeutics, including small molecule–small molecule, small molecule–biologics, and biologics–biologics. This chapter mainly introduces the recent progress of supramolecular-macrocycle-based nanomaterials in small molecule-small molecule combination therapy.

**Figure 9:**
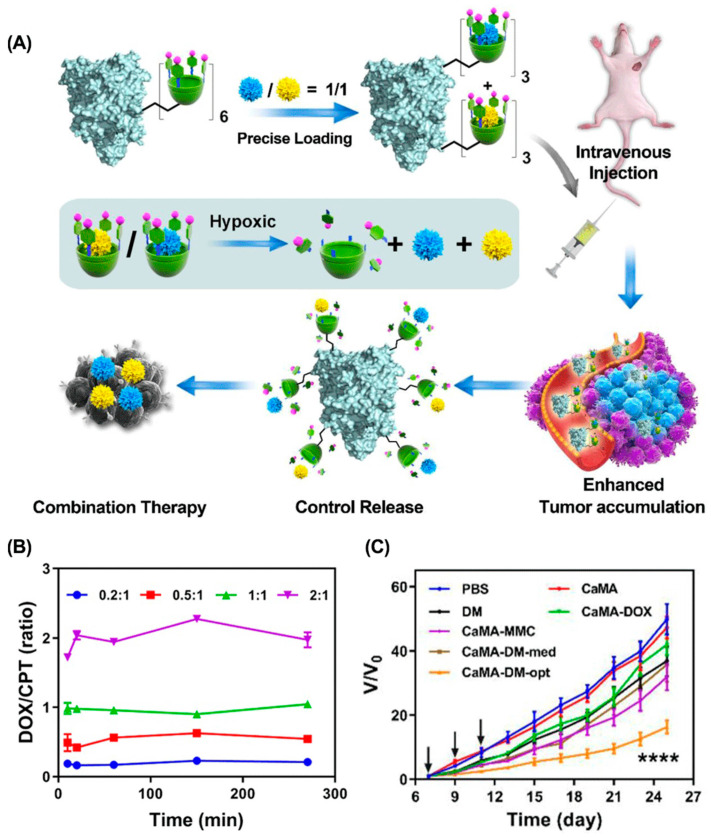
Calixarene-modified albumin for the ratiometric delivery of multiple drugs in combination chemotherapy. (**A**) Schematic illustrations of CaMA for combination chemotherapy. (**B**) The molar ratios of DOX to CPT released from CaMA-DC at different time points. (**C**) The therapeutic effect of CaMA-DM-opt on 4T1-bearing mice. (**** *p* < 0.0001). Reproduced from ref. [133]. Copyright 2022 Ivyspring International Publisher.

**Chemo-Chemotherapy.** In combination chemotherapy, the synergistic effect mainly depends on the concentration relationship, in which a certain proportion of the drug combination produces synergistic effects, while other proportions may be additive or antagonistic [131]. Therefore, ratiometric co-delivery of drug combinations in an optimized molar ratio to tumor tissues is essential for effective combination chemotherapy [132]. Macrocyclic molecules with defined chemical structures, predetermined cavity size, and characteristic binding affinity are ideal candidates for precise multidrug loading and ratiometric co-delivery. For example, our groups presented calixarene-modified albumin (CaMA) for the ratiometric delivery of multiple drugs in combination chemotherapy (Figure 9) [133]. Multiple hypoxia-responsive calixarenes (sulfonate azocalix(4)arene, SAC4A) were integrated into one albumin, which ensured the precise load and ratiometric co-delivery of multiple drugs to tumors via host–guest interaction. As it reached the hypoxic tumor microenvironment, SAC4A was reduced by reductase, leading to the spontaneous release of the drugs. By taking DOX and mitomycin C (MMC) as an example, delivering this drug combination at the optimal ratio via CaMA significantly enhanced tumor suppression compared to conventional cocktail therapy. Similarly, we developed a macrocyclic-amphiphile-based self-assembled nanoparticle and achieved the ratiometric co-delivery of PTX and NLG919 to tumor tissues [77].

**Figure 10:**
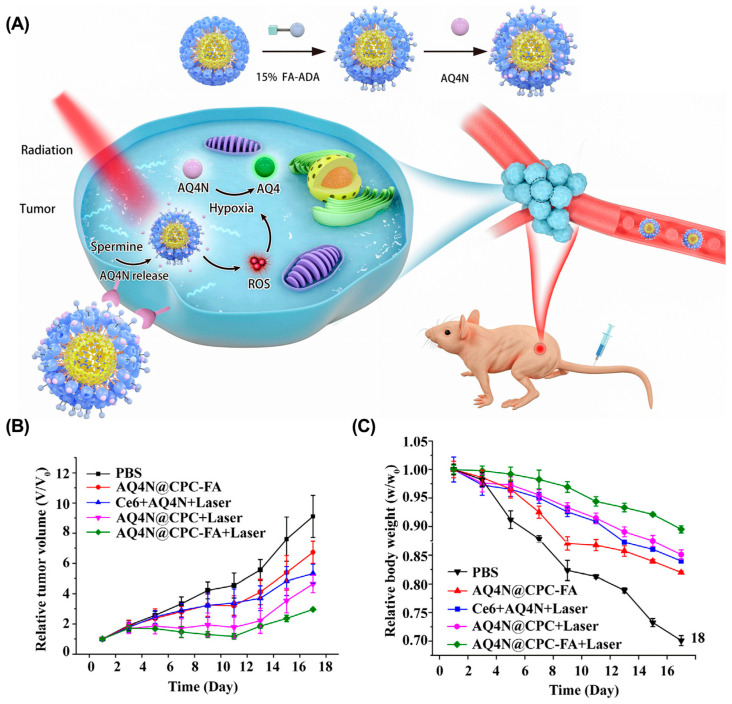
Supramolecular micelle for synergistic chemo-photodynamic therapy. (**A**) Schematic illustrations of AQ4N@CPC-FA for chemo-photodynamic therapy. (**B**) The tumor volume and the relative body weight (**C**) of mice after different treatments. Reproduced from ref. [135]. Copyright 2021 Elsevier.

**Chemo-photodynamic therapy.** Chemo-photodynamic therapy is a promising combination therapy strategy for cancer treatment, since (1) PDT usually has non-overlapping toxicity with chemotherapy; (2) PDT can overcome MDR by reducing the expression of P-gp to reduce drug efflux; and (3) ROS generated by PDT can be used as a stimulus to promote the drug’s release from the nanocarrier [134]. Loading chemotherapeutic agents and PS into nanocarriers is the most common method for combining these two therapies. For example, Wang et al. presented a supramolecular micelle to co-deliver Chlorin e6 (Ce6) and banoxantrone (AQ4N) for chemo-photodynamic therapy (Figure 10) [135]. Ce6 and AQ4N were loaded into the cavity of cucurbit(7)uril (CB(7)) and were precisely released in tumor tissues in response to overexpressed spermine. After irradiation, the hypoxic environment activated the prodrug AQ4N to chemotherapeutic AQ4, enabling synergistic chemo-photodynamic therapy. In another example, Xu et al. designed a ROS-responsive cyclodextrin-based polymeric micelle to co-deliver DOX and purpurin 18 (P18) [136]. After irradiation with a near-infrared laser, P18 in polymer micelles generated ROS, promoting DOX release and tumor cell apoptosis. Both in vitro and in vivo studies suggested that this strategy provided an effective method for the combination of chemotherapy and photodynamic therapy. Similarly, Yao et al. fabricated a WP6-based supramolecular polypeptide nanomedicine (BPC/DOX-ICG) for the co-delivery of DOX and indocyanine green (ICG), resulting in enhanced antitumor efficacy in an MCF-7/ADR tumor mouse model [137].

**Photothermal immunotherapy.** Photothermal therapy (PTT) is another promising cancer treatment strategy with the advantages of minimal invasiveness and high precision [138]. PTT utilizes photothermal reagents to absorb light, convert the light energy into local hyperthermia, disrupt cell membrane integrity, and induce tumor cell apoptosis or necrosis. In addition, PTT also promotes the release of damage-associated molecular patterns (DAMPs), including calreticulin (CRT), high mobility group box 1 (HMGB-1), and adenosine triphosphate (ATP), thus effectively triggering immunogenic cell death (ICD) and eliciting an antitumor immune response [139,140]. With this unique property, supramolecular-macrocycle-based nanomaterials are widely used in photothermal immunotherapy [141]. Nanogold is an ideal photothermal reagent with high photothermal conversion efficiency. For example, Yan et al. integrated gold nanoparticles into a supramolecular system and reported a gold nanorod-based nanosystem (GNR) for enhanced photothermal immunotherapy [142]. GNR was formed by layer assembly of γ-CD-crosslinked PEI on the surface of gold nanorods, and then the N6-methyladenosine demethylase inhibitor meclofenamic acid (MA) was loaded into the cavities of γ-CD to form GNR-CDP8MA. As it reached tumor tissues, the NIR-II induced the release of MA from γ-CD of GNR-CDP8MA, thus effectively decreasing the stability of PD-L1 transcripts. As a result, GNR-CDP8MA achieved effective photothermal immunotherapy in prostate cancer tumor-bearing (RM-1) mice. Similarly, Ping et al. reported a novel gold nanorod-based supramolecular nanomaterial (ANP/HSP) to disrupt the programming death-1/programming death ligand-1 pathway (PD-1/PD-L1) for enhanced photothermal immunotherapy [143]. The disruption of the PD-1/PD-L1 pathway synergized with PTT-induced ICD, resulting in significantly enhanced antitumor efficacy in B16F10 melanoma mice.

### 6.3. Supramolecular-Macrocycle-Based Combination Therapy in Overcoming Drug Resistance

**Figure 11:**
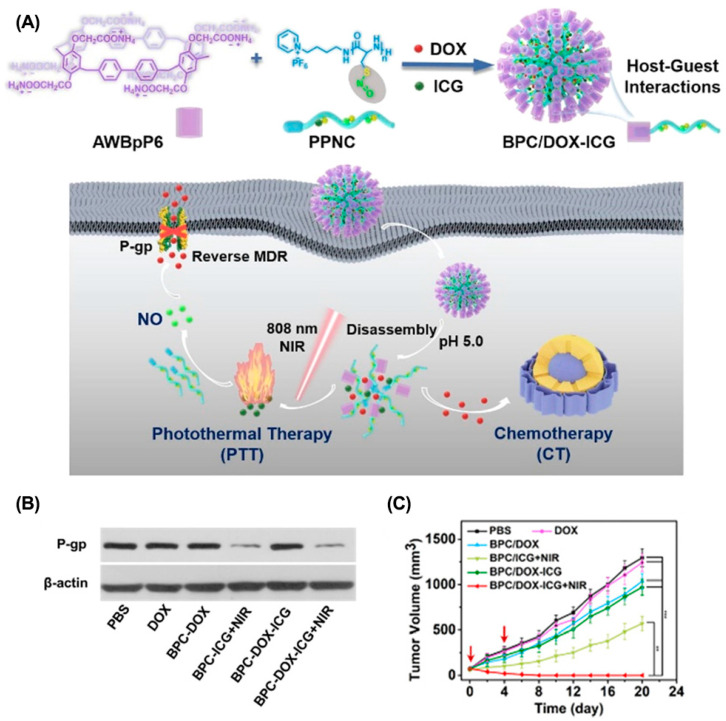
Supramolecular polypeptide nanomedicine for enhanced combination cancer therapy to overcome MDR. (**A**) Schematic illustration of the preparation of supramolecular polypeptide nanomedicine for combination cancer therapy. (**B**) Western blot analyses of the expressions of P-pg. (**C**) The tumor volume in each treatment group. Reproduced from ref. [137]. Copyright 2022 The Royal Society of Chemistry.

Multidrug resistance (MDR) refers to the ability of cancer cells to develop resistance to other anticancer drugs while being induced by a single anticancer drug during cancer therapy [144]. MDR is mainly associated with the overexpression of channel proteins on the cell membrane, such as p-glycoprotein (P-gp) [145]. As a typical ATP energy-dependent efflux pump, P-gp can efflux anticancer drugs to the extracellular TME, thereby reducing the antitumor efficacy of chemotherapy [146,147,148]. Therefore, co-delivering P-gp inhibitors with anticancer drugs is a promising strategy to reverse MDR. For example, Tian et al. developed a supramolecular assembly (SDDNMs) to reverse MDR based on the host–guest interactions between curcumin-modified β-CD and ferrocene-CPT [149]. After internalization, high levels of H_2_O_2_ efficiently disrupted host–guest complexation, leading to the rapid release of CPT and curcumin derivatives. As a result, SDDNMs effectively reversed MDR and greatly enhanced the antitumor efficacy of CPT in a B16F10 melanoma mouse model. In addition to p-gp inhibitors, low levels of nitrogen monoxide (NO) can down-regulate p-gp expression, reduce drug efflux, and alleviate MDR. Yao et al. constructed a supramolecular polypeptide nanomedicine to co-deliver ICG, DOX, and S-nitrosothiol (SNO, NO donors) for enhanced combination cancer therapy (Figure 11) [137]. After internalization, the nanomedicine disintegrated in response to acid and released the ICG and DOX. Upon irradiation, S-nitrosothiol efficiently generates NO due to PTT-induced hyperthermia. As a result, this strategy effectively reversed MDR and suppressed tumor growth in an MCF-7/ADR breast mouse model. In addition, cutting off the energy supply to P-gp is another strategy to reverse MDR [150]. Wu et al. reported a supramolecular assembly based on the host–guest complexation of β-CD with 1-adamantaneacetic acid to co-deliver DOX and adjudin (ADD), which is a mitochondrial inhibitor [151]. The controlled and sustained release of DOX and ADD in response to internal tumor acidity inhibited mitochondrial function and reduced ATP synthesis, thereby reducing the efficiency of P-gp.

**Table 1:** Advanced supramolecular-macrocycle-based nanomaterials in cancer treatment.

**Types**	**Macrocycles**	**Formulations**	**Payloads**	**Tumor**	**Refs**
Chemotherapy	WP5	Supramolecular vesicles	DOX	MCF-7	[86]
Chemotherapy	WP5	Supramolecular vesicles	DOX	HpeG2	[87]
Chemotherapy	WP5	Vesicles or micelles	DOX	HpeG2	[88]
Chemotherapy	β-CD	LbL films	DOX	A549	[89]
Chemotherapy	β-CD	Supramolecular nanoparticles	DOX	4T1	[90]
Chemotherapy	WP5	Supramolecular nanoparticles	DOX	CT26	[96]
PDT	β-CD	Supramolecular nanoparticles	TPP	4T1	[107]
PDT	β-CD	Supramolecular organic framework	TPP	4T1	[108]
PDT	GC5A-12C	Supramolecular nanoparticles	AlPcS4	4T1	[111]
PDT	SC4A	Supramolecular nanoparticles	TPE-PHO	4T1	[112]
PDT	WP6	Supramolecular photosensitizer system	MB	HepG2	[116]
PDT	WP5	Supramolecular metallodrug micelles	Curcumin	B16	[118]
PDT	β-CD	Supramolecular nanoparticles	Ce6	4T1	[119]
PDT	β-CD	Supramolecular vesicles	Ce6	4T1	[121]
Chemo-chemotherapy	SAC4A	Calixarene-modified albumin	DOX and MMC	4T1	[133]
Chemo-chemotherapy	QAAC4A-12C	Supramolecular nanoparticles	PTX and NLG919	4T1	[77]
Chemo-photodynamic therapy	CB(7)	Supramolecular vesicles	Ce6 and AQ4N	MCF-7	[135]
Chemo-photodynamic therapy	β-CD	Supramolecular micelles	DOX and P18	4T1	[136]
Chemo-photodynamic therapy	WP6	Supramolecular polypeptide nanomedicine	DOX and ICG	MCF-7/ADR	[137]
Chemo-chemotherapy	β-CD	Supramolecular micelles	CPT and Cur	B16	[149]
Combination therapy	AWBpP6	Supramolecular polypeptide nanomedicine	DOX and SNO	MCF-7/ADR	[137]
Chemo-chemotherapy	β-CD	Supramolecular nanoparticles	DOX and ADD	MCF-7/ADR	[151]
Photothermal immunotherapy	γ-CD	Gold nanorod-based supramolecular nanomaterial	MA	RM-1	[142]
Photothermal immunotherapy	β-CD	Gold nanorod-based supramolecular nanomaterial	HSP-Cas9 plasmid	B16F10	[143]

## 7. Conclusions and Perspectives

With the rapid development of supramolecular chemistry, supramolecular macrocycle-based nanomaterials with diverse functions have shown great potential in biomedical applications. Because of their dynamic, reversible host–guest interactions and sensitivity to abnormal tumor indicators (acidic, redox, ATP, and hypoxic), supramolecular-macrocycle-based nanomaterials have been widely explored in chemotherapy, photodynamic therapy, and combination therapy for cancer treatment. Despite the remarkable success, the clinical benefits of supramolecular-macrocycle-based nanomaterials still face many obstacles, including (1) the introduction of chemical groups to modify the host or guest molecules is often accompanied by complex chemical synthesis and purification processes, and the resulting new host or guest molecules may have toxic effects; (2) dynamic and weak non-covalent interactions may lead to leakage of immature drug into the blood circulation, resulting in poor therapeutic efficacy and severe side effects; (3) the self-assembled nature of supramolecular-macrocycle-based nanomaterials may result in batch-to-batch variability.

For the better development of supramolecular-macrocycle-based nanomaterials in biomedical applications, the following aspects need to be considered in the future: (1) the potential toxicity of the introduced modification groups should be thoroughly analyzed to avoid unnecessary toxicity; (2) new supramolecular hosts with better biocompatibility should be developed; (3) diagnostic and imaging capabilities of supramolecular nano-systems should be enhanced for more accurate therapy; and (4) the assembly and preparation process should be optimized to reduce batch variance.

## Data Availability

Not applicable.
